# Operating procedures, risk management and challenges during implementation of adaptive and non-adaptive MR-guided radiotherapy: 1-year single-center experience

**DOI:** 10.1186/s13014-021-01945-9

**Published:** 2021-11-14

**Authors:** Helena Isabel Garcia Schüler, Matea Pavic, Michael Mayinger, Nienke Weitkamp, Madalyne Chamberlain, Cäcilia Reiner, Claudia Linsenmeier, Panagiotis Balermpas, Jerome Krayenbühl, Matthias Guckenberger, Michael Baumgartl, Lotte Wilke, Stephanie Tanadini-Lang, Nicolaus Andratschke

**Affiliations:** 1 Department of Radiation Oncology, University Hospital Zurich and University Zurich, Rämistrasse 100, 8091 Zurich, Switzerland; 2 University of Zurich (UZH), Rämistrasse 100, 8091 Zurich, Switzerland; 3 Department of Diagnostic and Interventional Radiology, University Hospital Zurich and University Zurich, Rämistrasse 100, 8091 Zurich, Switzerland

**Keywords:** MR-guided radiotherapy, Online-adaptive radiotherapy, Image-guided radiotherapy, Risk analysis, SBRT

## Abstract

**Background:**

Main purpose was to describe procedures and identify challenges in the implementation process of adaptive and non-adaptive MR-guided radiotherapy (MRgRT), especially new risks in workflow due to the new technique. We herein report the single center experience for the implementation of (MRgRT) and present an overview on our treatment practice.

**Methods:**

Descriptive statistics were used to summarize clinical and technical characteristics of treatment and patient characteristics including sites treated between April 2019 and end of March 2020 after ethical approval. A risk analysis was performed to identify risks of the online adaptive workflow.

**Results:**

A summary of the processes on the MR-Linac including workflows, quality assurance and possible pitfalls is presented. 111 patients with 124 courses were treated during the first year of MR-guided radiotherapy. The most commonly treated site was the abdomen (42% of all treatment courses). 73% of the courses were daily online adapted and a high number of treatment courses (75%) were treated with stereotactic body irradiation. Only 4/382 fractions could not be treated due to a failing online adaptive quality assurance. In the risk analysis for errors, the two risks with the highest risk priority number were both in the contouring category, making it the most critical step in the workflow.

**Conclusion:**

Although challenging, establishment of MRgRT as a routinely used technique at our department was successful for all sites and daily o-ART was feasible from the first day on. However, ongoing research and reports will have to inform us on the optimal indications for MRgRT because careful patient selection is necessary as it continues to be a time-consuming treatment technique with restricted availability. After risk analysis, the most critical workflow category was the contouring process, which resembles the need of experienced staff and safety check paths.

**Supplementary Information:**

The online version contains supplementary material available at 10.1186/s13014-021-01945-9.

## Background

Verification of patient and tumor position is a prerequisite for accurate delivery of radiation dose to the target volume. Image guided radiation therapy (IGRT) has undergone a significant transformation in the past: from skin marks over simple radiographic films to electronic portal imaging devices using a treatment-machine mounted kV-source and a flat panel detector or even in-room computed tomography (CT) on rails [1]. Most notably, introduction of Cone-beam CT (CBCT) and CT on rails introduced the possibility to visualize the tumor immediately before each treatment fraction throughout the body and allowed for the adoption of high-dose radiation therapy. Image guidance allowed for significant reduction of setup margins and has been shown to improve clinical outcomes in numerous tumor entities [2]. The latest and potentially practice changing development in IGRT is the advent of MR-guided radiotherapy (MRgRT) through novel hybrid linear accelerators with built-in MR imaging capabilities[3]. The major advantages of MRgRT are the superior soft tissue contrast compared to CT, real-time imaging for accurate tumor tracking and finally the possibility for on-table target and organ-at-risk (OAR) adaptation with subsequent re-planning to adapt to the anatomy-of-the-day. Plan adaptation during the course of radiotherapy has been shown to be advantageous in cases where changes in anatomy of either OAR or tumor are likely, for example in head and neck, lung or prostate cancer [4–7]. This technical evolution offers a significant potential to improve the therapeutic ratio by better tumor visualization and targeting as well as by novel adaptive dose optimization concepts raising the expectations for paradigm change in radiation oncology [8].

Since the introduction of the first hybrid MR-Radiotherapy worldwide [9], implementation of MRgRT continues at multiple radiation centers and relevant work has been published on the adaptive workflow and its clinical benefits [10–12]. Still, introduction of such a new technology and the paradigm change with daily adaptation represents a major challenge for implementation into clinical practice. The general adoption by the community will not only benefit from clinical outcome analysis, but also from reports on clinical implementation, identification of early-phase pitfalls, improvements of workflows and approaches to quality assurance and risk assessment.

The main purpose of this report was to evaluate the workflow processes for the clinical implementation of adaptive MR-guided radiotherapy (MRgRT) with a dedicated hybrid MR linear accelerator (MRIdian System; ViewRay Inc. Oakwood Village, OH), by identifying critical steps, main challenges and potential risks.

## Methods

### Data and records review, statistical analysis

Installation of the system started in October 2018 and in December 2018 the MR-LINAC passed the acceptance testing procedure (ATP). The commissioning phase (January-March 2019) was used to get familiar with the new imaging modality in combination with radiotherapy as well as to conduct a dedicated quality assurance procedure, tailored to the new technology and implement the interdisciplinary clinical workflow.

All patients included in this retrospective analysis were treated with MRgRT from April 2019 until end of March 2020. The technical specifications of the MRIdian System (ViewRay©), a hybrid linear accelerator with a 0.35 T magnetic resonance imaging (MRI) system, have already been described previously [3]. The analysis was approved by the cantonal ethics committee Zurich (BASEC-Nr. 2018-01794).

The clinical information system, data records from the ViewRay software, as well as radiation therapy plans were reviewed to obtain the information presented in the results section. In addition, quality assurance (QA) information was retrieved from our QA records performed for all process steps of MRgRT. All follow-up data until 12th of June 2020 were considered and included. Acute toxicities were graded according to CTCAE, version 5.0. Data analysis was performed using descriptive statistics, median values and ranges have been calculated where necessary. All statistical analyses were performed in Excel (Microsoft Corporation, Redmond, WA, USA).

### Screening, simulation, treatment planning and adaptive planning workflow

Patient screening for potential treatment on the MR-LINAC was performed at their first outpatient visit. Eligibility criteria were defined in a structured screening survey as follows: ability to undergo an MRI without any contraindication (no metallic devices, piercing or tattoos or cardiac pacemakers not compatible with MRI, no claustrophobia) and to lie in the treatment position for approximately 60 min. In case of tumors that generally move with respiration (tumors in the lung or the abdomen), patients had to be able to perform breath-hold for a minimum of 17 s to achieve a qualitative image suitable for simulation and adaptation and to minimize delivery time in breath-hold. Patients fulfilling all these criteria underwent the simulation process. The screening checklist is available in the Supplement.

MR simulation at the MR-LINAC, consisted of a scan time optimized high-resolution scan and a cine gating scan to evaluate stability and tracking quality for breath-hold-cases and a subsequent computed tomography (CT) scan in treatment position, to obtain information on electron density for dose calculation. Breath-hold commands were given per audio-system. No visual coaching system was installed at this time. MR-compatible immobilization devices were used where needed, e.g. thermoplastic mask for treatment of head-and-neck-patients (Posicast®-Plus PR5 (CIVCO Medical Solutions) Coralville, Iowa (USA) or a dedicated setup with vacuum bags for SBRT (Vac-Lok™ Cushions (CIVCO Medical Solutions) Coralville, Iowa (USA)). Scanning time of the different default TRUFI-Scans varied between 17 s and 3 min, in dependence of site, field of view and resolution. For breath-hold sequences, standard scan times were 17 and 25 s.

After simulation, target volume and OAR delineation as well as treatment planning were performed within the ViewRay planning system. For dose-calculation a 2 mm dose grid in case of stereotactic treatments and a 3 mm dose grid for all other treatments was used. The magnetic field was taken into account in the calculation and the uncertainty set to 1%.

**Fig. 1:**
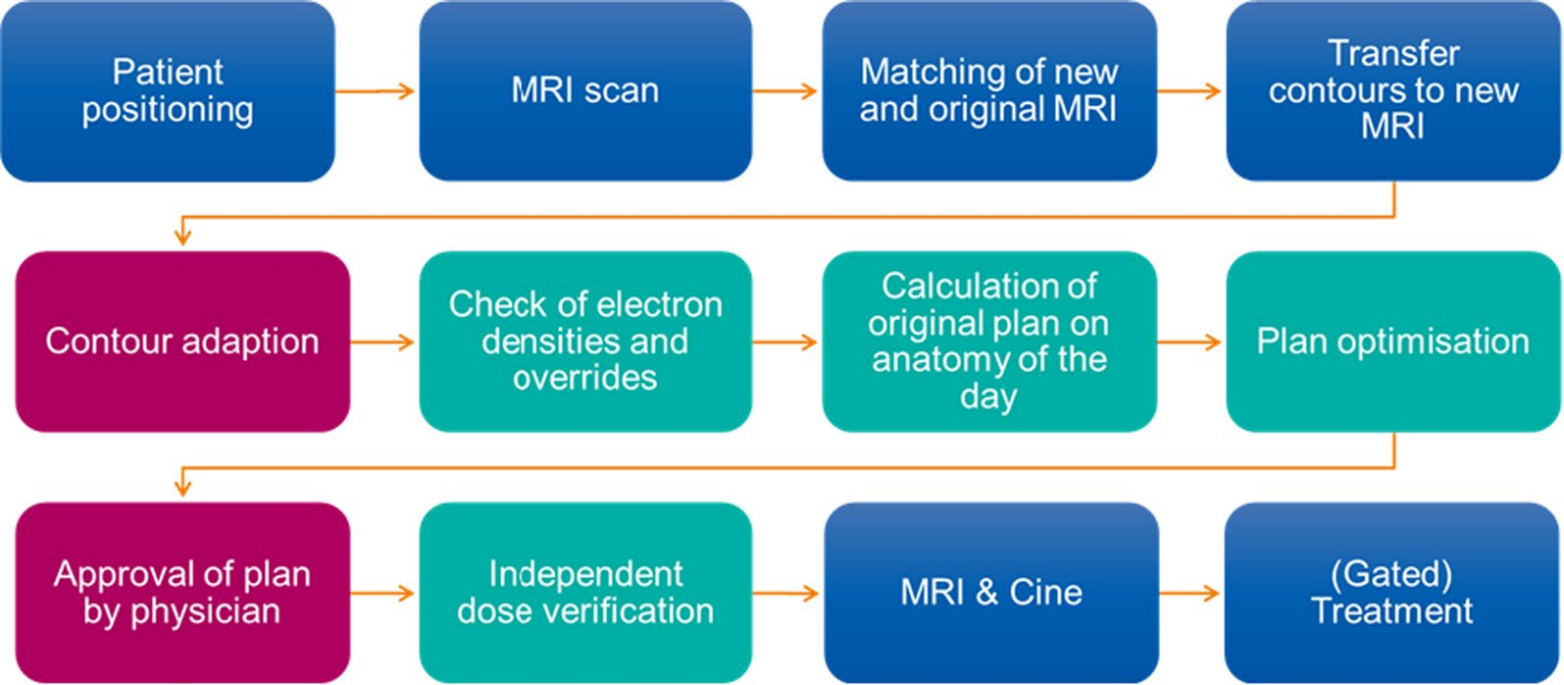
Adaptive workflow for a treatment at the MR-LINAC. Tasks performed by RTTs are blue, by physicians depicted in red and by physicists or dosimetrists in turquoise

A core team defined our workflow for daily online adaptive radiotherapy (o-ART) after site visits to other MR-LINAC centers and in-house training, before the actual start of the clinical treatment program. Different approaches of clinical use of MRgRT were evaluated and finally tailored to the local needs and specifications of our center. Our final adaptive workflow is displayed in Fig. 1.

For contour adaptation, all structures created during the initial base plan were transferred to the newly acquired MRI and registered by deformable image registration except the Target Volumes. The nearby OARs were re-contoured within a 2 cm isotropic ring around the PTV because of the non-perfect deformable registration. Density corrections of air-filled structures were made based on the anatomy of the day [12]. The rules created during the initial treatment planning process were applied to create the PTV and help structures as needed for the optimization. Plan optimization was conducted with the same optimization objectives as the original plan. In the first pass, a weight optimization was performed. There segment number and shape remained the same and only the weighting of each of these segments was optimized. If this plan did not meet the defined objectives constraints, a full optimization was calculated, where the segment shapes were also optimized [12]. If the treatment was delivered using gating the GTV was extended by 3–5 mm and this was used as a gating window. We allowed up to 5% of the target to be outside this gating window.

### Quality assurance for adaptive planning

For every adapted plan, a patient-specific QA was performed before treatment delivery with an independent Monte-Carlo dose calculation algorithm and gamma analysis (integrated in the ViewRay® system). The passing criteria were: a global gamma value > 95% with maximum of 3% dose difference and 2 mm distance to agreement.

The plan was additionally verified with an independent point dose calculation using RadCalc V6.3 (LifeLine Software Inc.), which does not take the magnetic field into account. A dose difference up to 10% was accepted. For lung cases a larger dose deviation was accepted if the integral dose (calculated as sum of the product of the field size, output factor and the percent depth value calculating over each segment) was in agreement (< 10% deviation) with the initial plan.

Additionally, for the first 20 patients for each tumor site, all the adapted plans were verified after treatment delivery by default with the Delta 4 phantom. After this phase, the first adapted plan per patient was verified using the Delta4 phantom, as well as plans that had a high complexity (calculated according to [13]. Results were evaluated using a global gamma agreement score with a 3% dose difference, a 2 mm distance to agreement and a 20% dose threshold.

### Risk analysis

**Fig. 2:**
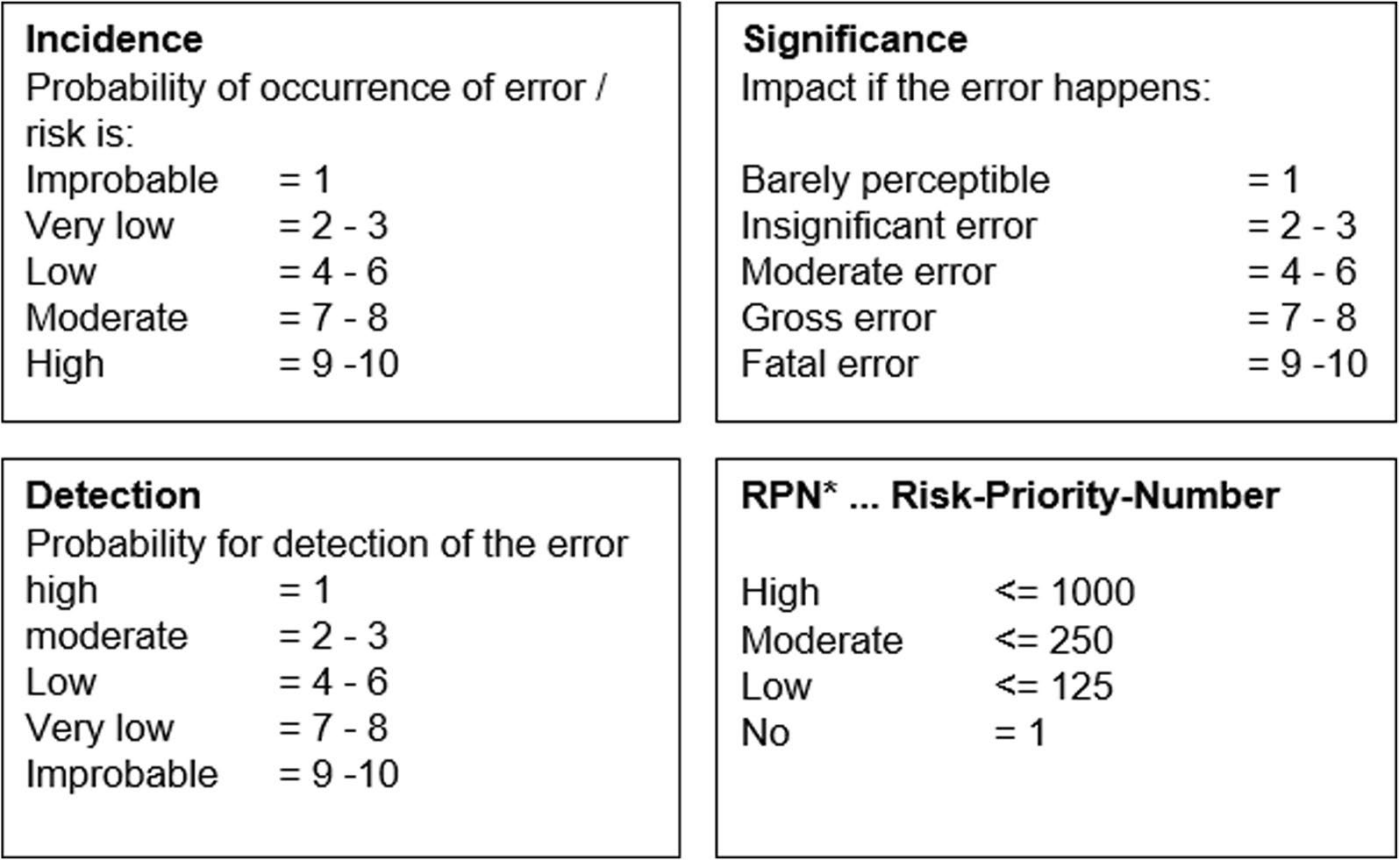
Risk categories as defined by the FMEA risk assessment methods

As a novel instrument to assess and adjust our procedures of MRgRT, a risk analysis (Failure Mode and Effects analysis FMEA) was performed to identify potential risks and classify their likelihood of occurrence, detection and impact on treatment quality and safety. For each described risk, a Risk Priority Number (RPN) was assessed [14, 15] (Fig. 2).

## Results

### Patient screening, compliance and treatment aspects

The core team had initially defined beneficial indications for MRgRT based on the following criteria: (a) anticipated benefit from enhanced imaging and motion management, (b) anticipated benefit from daily adaptation, ((c) prospective studies to define benefit of online-adaptive radiotherapy (o-ART).

Between April 8th 2019 and 31st March 2020, 150 patients were considered suitable for treatment at MR-LINAC of whom 111 patients were finally treated at MR-LINAC. Patient and treatment characteristics are summarized in Tables 1 and 2.

**Table 1:** Patient and general treatment characteristics

Total patient number	111
Male n (%)	81 (73)
Female n (%)	30 (27)
Age, median (range)	67 (27–88)
Pacemaker n (%)	4 (4)
Total treatment courses	124
SBRT n (%)	94 (76)
Mixed courses (Boost at MRIdian) n (%)	17 (14)
Conventional fractionation (range)	13 (10)
Re-Irradiation*; n (%)	2 (2)
Treatment discontinuation or interruption; n (%)	2 (2)

^*^Definition: at least overlap of the 50% isodose

**Table 2:** Technique distribution per treatment site

Site	Total treatment courses n (%)	SBRT	Mixed courses (Boost at the MR-LINAC)	Conventional fractionation exclusively at the MR-Linac
Treatment diagnosis/site; n (%)	124			
*Head and neck*	9 (7)	0	2	7
*Lung*				
Mediastinum	3 (2)	2	0	1
Lung	13 (10)	12	0	1
*Abdomen*				
Liver	24 (19)	19	4	1
Pancreas	8 (7)	8	0	0
Abdominal nodes	8 (7)	8	0	0
Adrenal gland	7 (6)	7	0	0
Kidney	4 (3)	4	0	0
*Pelvis*				
Pelvis	16 (13)	13	3	0
Prostate	18 (15)	9	8	1
*Bone*	10 (8)	10	0	0
*Others* (2 cardiac SRS, 1 sarcoma of the limb, 1 peripheral nerve sheath tumor at the lumbar spine)	4 (3)	2	0	2

**Fig. 3:**
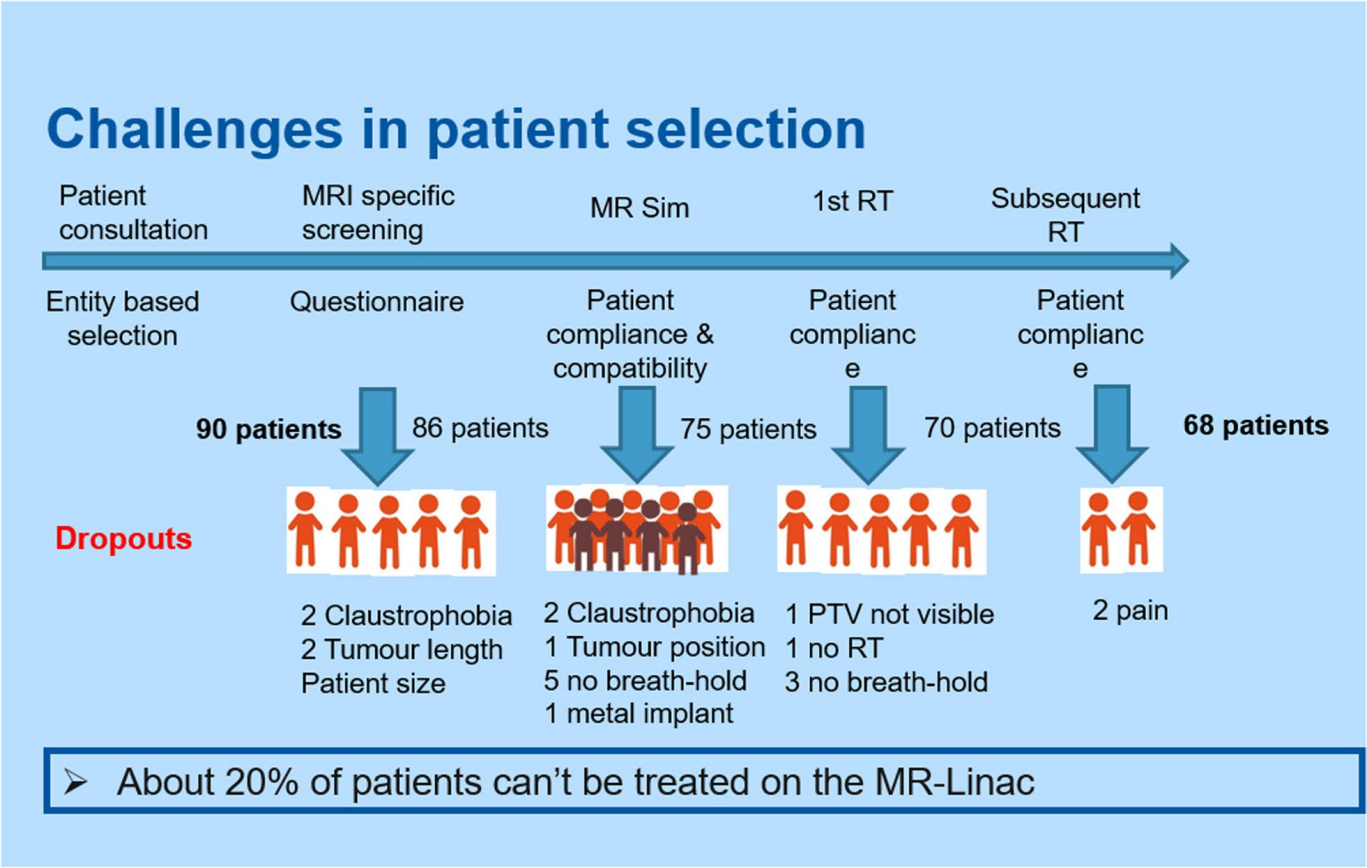
Dropouts during selection process—from screening to treatment

Reason for and time point of dropout were recorded to evaluate quality of patient selection. Dropout reasons were mainly clinical (13.3%, Fig. 3) and in part technical (patient positioning, gating correlation), despite detailed upfront screening. After treatment start, dropout rates were low. Only two treatment courses (1.8%) had to be terminated prematurely. One patient experienced a pronounced acute toxicity under SBRT of the prostate with dermatitis, proctitis, cystitis and urinary tract obstruction, all grade 2 and treatment was discontinued after 4 of 5 planned fractions with a total dose of 29 Gy. The other patient needed to switch to conventional LINAC treatment for the inability to lie in treatment position after two fractions. For details of dropout distribution path see Fig. 3.

75% of the treatment courses were treated with SBRT, mostly applied to targets located in the upper abdomen (24%) and liver (20%). Details of irradiation techniques and site distribution are summarized in Table 2.

**Fig. 4:**
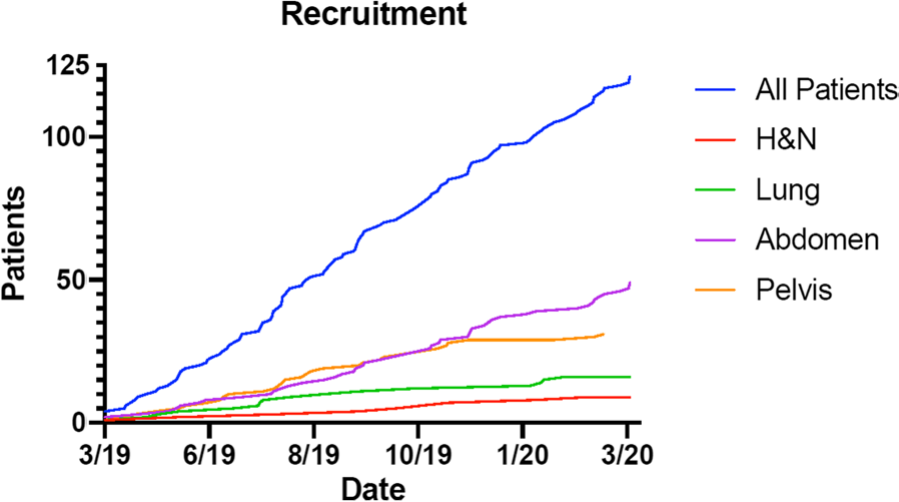
Recruited patients for treatment at the MR-Linac with numbers for all patients in total. Separately displayed are the four different areas ‘H&N = head and neck’, ‘abdomen’, ‘lung’ and ‘pelvis’

Site distribution as shown in Table 2 shifted over time. Recruitment of patients treated in the abdominal region (pancreas, abdomen, liver, adrenal gland and kidney) increased, whereas there was a trend for slower recruitment for treatment of the lung and head-and-neck (shown in Fig. 4).

Most patients (82%) were online adapted. All patients treated with conventional fractionation and without online adaptation had a weekly re-planning based on the new imaging during therapy, so called offline weekly adaptation, to ensure the highest plan precision. 62% of SBRT were performed in breath-hold. A summarizing table with details of planning characteristics and dose in the different groups can be found in the supplement.

### Quality assurance of daily adaptive plans

Four out of 382 plans (1%) did not pass one of the QA steps and were therefore not treated with a re-optimized plan but instead with the initial treatment plan: in two cases, RadCalc dose deviation was above our tolerance of 10% and in the other two the independent Monte Carlo dose verification was out of tolerance. All plans verified with the Delta 4 passed our Gamma criteria with a value > 95% with one exception. The one plan not passing was a head and neck case with a not perfect phantom setup.

### On-Table time

On-Table time was evaluated consequently throughout the first 9 months for all patients with daily adaption (n = 85). The time of patient set-up, acquisition of the first image, contouring, plan adaption, treatment time and getting of the treatment couch were separately recorded. We included 10 thoracic, 32 pelvic and 43 abdominal treatments with a variety of fractionation schemes. The median treatment time was 57 min (range 20–110), from first image until beam off. Differences between treatment sites are described in Table 3. Difference in treatment times between pelvis and abdomen were mainly due to contouring and beam delivery time. Remarkably, during the first three months, median treatment time was 52 min, followed by 58 min during the next three months and 61 min during last 3 months. The longest on-table times were for two abdominal cases, mainly caused by complex positioning and offset, low correlation in breath-hold gating and complex contouring. The two fastest adaptive cases, both pelvic nodes with no critical OARs adjacent and no gating.

**Table 3:** Mean treatment time depending on treatment site

	Mean treatment time (min)	Patients (n)
Overall	57 (20–110)	85
Thoracic	56 (33–80)	10
Contouring	4	
Delivery	15	
Abdominal	61 (36–110)	43
Contouring	13	
Delivery	15	
Pelvic	53 (37–93)	32
Contouring	11	
Delivery	10	

### Risk analysis

As the introduction of new and different workflow steps with a novel technology, not readily familiar to the community, carries significant uncertainties and potential risks, we decided to implement a risk analysis strategy, which consisted of a prospective risk assessment of the different workflow steps.

This was performed in a multi-disciplinary team of radiation oncologists, medical physicists and radiation therapists. In total, we identified 21 risks in six different categories: patient setup, imaging and matching, contouring, adaptive planning, quality assurance and overall process. Detailed risks are listed in the Supplement. The two risks with the highest Risk Priority Number (RPN) were both in the contouring category, making it the most critical step in the workflow. Nevertheless, due to a vigorous implementation of a quality assurance concept and a dedicated workflow with individual checkpoints all risks were considered to be in the low risk category (Fig. 1).

The two major risks with an RPN of 42 were:

First, a change in the auto-segmentation of the skin contour possibly leading to a non-representative and incorrect dose calculation. Two measures were identified to check the skin contour. First, an item was added to the adaptive checklist to visually check the correctness of the automatic contouring the skin contour. Second, an automatic check was implemented to compare the equivalent path length for each field between the original and the adapted plan. In addition to an automated cross-check of the auto-segmentation, this step also helped to identify changes in the patient’s anatomy due to different positioning or weight loss. In case of a difference of more than 2 cm, the responsible physicists visually inspected the two plans for anatomical reasons of the difference.

The second major risk was a wrong assignment of tissue densities during the contouring process, which may lead to a difference between the actual and the calculated dose distribution. The counter-measures taken were a visual check of the densities using a checklist and an automatic comparison of different dosimetric measures between the original plan and adapted plan such as the total number of MU, the product of MU times irradiated area and the same product corrected by the equivalent path length.

After 3 months of clinical operation, the risk analysis was re-evaluated in the same multi-disciplinary team and one additional risk was added connected to the overall workflow. Due to new staff members being trained on the MR-LINAC and many visitors during this time, the control room during adaptions was rather busy. Therefore, the inattentiveness during the adaptive process was rated with an RPN of 24 (third highest value) and it was agreed to generate a quiet atmosphere during the adaptions and allowing a maximum of 4 persons in the control room during these procedures.

## Discussion

We herein report procedures and main challenges in the implementation process of adaptive and non-adaptive MRgRT on a linear accelerator-based hybrid MRgRT system introduced at our institution in April 2019 (MRIdian System; ViewRay Inc.). As this was considered a complex process with several new features like integrated MR imaging capabilities as well as the online on-table adaptive process, our focus from the beginning was on patient screening and compliance, QA of the adaptive workflow and risk assessment.

In order to apply MRgRT to cases with highest expected benefit and taking into account the time and high resources needed, patient selection is of utmost importance. With the implemented MR screening process, we could successfully filter patients not suitable for MRgRT and only a 1.8% dropout rate during treatment itself was observed. Most screening dropouts were observed at MR simulation (10%), when the workflow could be tested in reality, and patients dropped out mostly for not being able to follow breath hold commands despite screening and information beforehand.

Claustrophobia and anxiety are well known barriers in MRI imaging and several interventions were developed for their reduction and relief [16]. Careful anamnestic preselection was sufficient as screening and resulted in no dropouts due to patients discomfort or new claustrophobia induced during therapy. A good feasibility for MRgRT with only 29% of patients complaining of the MRI has already been described in literature [17].

In addition, with increasing experience and the respective analyses of the benefit of online adaptive radiotherapy [12, 18, 19] the indications for MRgRT treatment shifted towards predominantly abdomino-pelvic treatments and a slower recruitment for thoracic tumors. Therefore, the number of treatments in the upper abdomen and pelvis increased over time as indicated in Fig. 3 and represents with 41% the main anatomical location of our treatment courses.

**Fig. 5:**
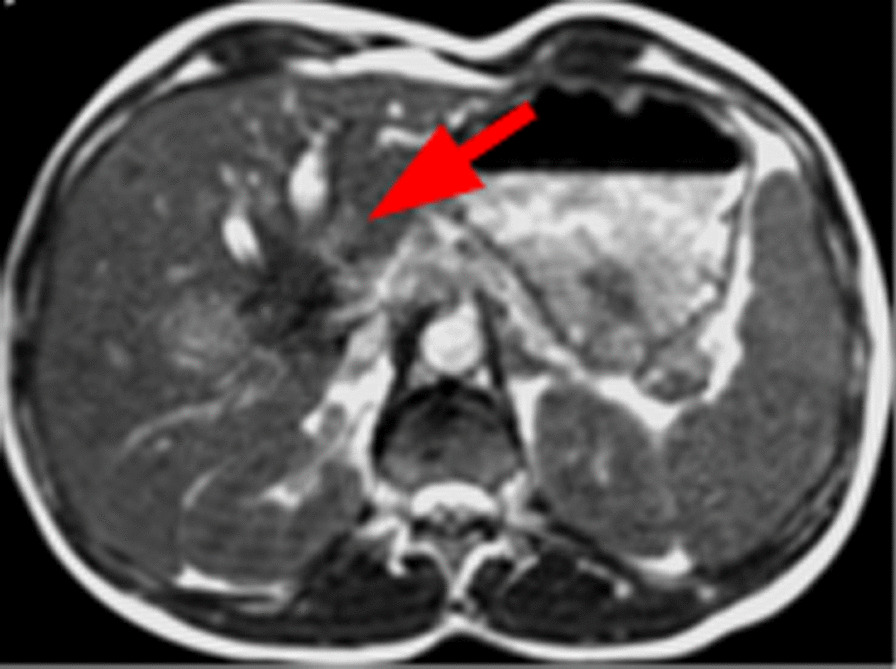
Appearance of a cholangiocellular carcinoma in the simulation sequence at the MR-Linac. Red arrow signs to the hypo-intense tumor region

Although MRgRT is predominantly used to deliver SBRT, to exploit the superior soft tissue visualization for target localization we explored an additional indication: delivery of the conventionally fractionated boost series at the MR-LINAC, while treating the main series with elective regions at the conventional CT-LINAC. These were for macroscopic recurrences of the prostate bed and cholangiocellular carcinoma (CCC). An example for images of a CCC in MR simulation is shown in Fig. 5.

In addition, MgRT of head-and-neck-patients is not common yet. Of this cohort, seven patients were HNC cases, treated within a clinical study, which is still recruiting (ClinicalTrials.gov Identifier: NCT03972072). Technical considerations and feasibility of high-quality-planning for these patients at the machine were recently presented [20, 21].

Monitoring of on-table time revealed that the major variance was related to the adaptation of the contours mean contouring time increased with anatomical complexity of tumor site (Table 3). Any significant improvement in this adaptation step is only to be expected with improved auto-segmentation methods and investigations on the dosimetric impact of the contouring of OAR and density overrides. Compared to other institutional experiences within Europe our on-table time is longer than in some centers [22, 23]. Klüter et al. reported a mean on-table time of 40 min for gated treatments at their institution. However, they did not perform online plan adaptions. Another institution reported 45 min on-table time for prostate cases [17]. As we did not analyze our prostate cancer treatments separately, a direct comparison is not possible. Yet, differences in the per institution implemented individual workflows and regulatory requirement for quality assurance might in part also explain the observed difference.

Unexpectedly, our mean on-table time increased over the first 9 months from 52 to 61 min despite growing experience and routine. This may have several reasons. In the beginning, a fixed core team was responsible for the pre-implementation training and workflow development as well as for the online adaptive re-planning in the first months. This not only ensured constant process quality of the overall adaptive workflow, but also within the treatment of an individual patient level. This changed, as with growing confidence of the team and increasing patient numbers, we involved new staff and trained them on site accordingly. Additionally, the number of abdominal cases grew over the time (Fig. 3).

For conventionally X-ray based delivered IGRT, GTV-definition has been shown to have the highest potential for error due to its high inter-observer variability and uncertainty in imaging, while setup errors (random or systematic) are the most probable reason for incorrect delivery [24]. On top of these well-known factors, MRgRT adds new levels of complexity with the integrated MR imaging, added QA measures and especially with the adaptive planning process.

Therefore, in addition to target delineation and setup errors identified as most critical risks by van Herk [24], our risk analysis revealed new potential risks, that need to be taken into account with online adaptive MRgRT. Experienced radiation oncologists and strong agreements about workflow and contouring in the team, as well as regular training, were necessary to minimize these risks. By performing this risk analysis, precise checklists were developed and staff was trained for the crucial workflow steps.

The doses applied and the implemented regimens for MRgRT are based on treatment experience at the conventional LINAC. In the beginning of image-guidance and tracking, with usage of CBCT or fiducials CTV to PTV margins were reduced [25, 26]. The new additional information due to MR imaging has to be investigated closely before changing treatment concepts due to the adapted technique.

We are aware of the limitations of this analysis, as it is based on the experience of one dedicated hybrid MR-Linac system and reflects a single-center experience with its institutional specifications and peculiarities, which may only be translated in limited terms to other institutions. Nevertheless, we consider our analysis relevant to the community, as we investigated our patient screening and compliance, adaptive QA and on-table time with regards to the different workflow steps and applied a dedicated risk analysis to develop a safe process for daily online adaptive MRgRT. Although some of the first sites worldwide already published their experiences and workflows with MRgRT [27–29], this is one of the first implementation report on the individual process steps of the on-line adaptive workflow on a linear accelerator based MR hybrid system with a specific focus on patient selection and risk management.

## Conclusion

Although challenging, establishment of MRgRT as a routinely used technique at our department was successful for all sites and daily o-ART was feasible from the first day on. However, ongoing research and reports will have to inform us on the optimal indications for MRgRT because careful patient selection is necessary as it continues to be a time-consuming treatment technique with restricted availability. After risk analysis, the most critical workflow category was the contouring process, which resembles the need of experienced staff and safety check paths. Scientific collaboration between centers performing MRgRT and reports of institutional experiences will contribute to increase the evidence and practicability for this technique.

## Supplementary Information

**Additional file 1.** Screening checklist, detailed risk analysis, special aspects of treatment sites (liver, bone, prostate/pelvis, head and neck), quality assurance of the machine.

## Data Availability

The datasets analysed during the current study are not publicly available due to ongoing analyses, but are available from the corresponding author on reasonable request.

## Abbreviations

ATP: Acceptance testing procedure
CT: Computed tomography
CBCT: Cone-beam CT
CCC: Cholangiocellular carcinoma
IGRT: Image guided radiation therapy
MR: Magnetic resonance
MRI: Magnetic resonance imaging
MRgRT: MR-guided radiotherapy
QA: Quality assurance
OAR: Organ at risk
o-ART: Online adaptive radiotherapy
RPN: Risk priority number
SBRT: Stereotactic body irradiation
