# Supplementary material for: Operating procedures, risk management and challenges during implementation of adaptive and non-adaptive MR-guided radiotherapy: 1-year single-center experience
Source: Radiat Oncol. 2021 Nov 14;16:217. doi: 10.1186/s13014-021-01945-9 (PMC8591958; doi:10.1186/s13014-021-01945-9)
Supplement: Supplementary file 1 — Additional file 1. Screening checklist, detailed risk analysis, special aspects of treatment sites (liver, bone, prostate/pelvis, head and neck), quality assurance of the machine. [file 13014_2021_1945_MOESM1_ESM.docx]

**Supplementary Material:**

**First Year - Single center clinical experience with MR-guided radiotherapy: from head to toe**

Content

[Screening Checklist to reduce drop-outs 2](#_Toc71296959)

[Risk analysis 3](#_Toc71296960)

[Special aspects of treatment sites 6](#_Toc71296961)

[Liver 6](#_Toc71296962)

[Bone 7](#_Toc71296963)

[Prostate/Pelvis 7](#_Toc71296964)

[Head and neck 8](#_Toc71296965)

[Quality assurance of the machine 8](#_Toc71296966)

# Screening Checklist to reduce drop-outs

May we ask you to answer the following questions **(Where applicable please underline)**

| Last name, First name |  | Height (cm) |  |
| --- | --- | --- | --- |
| Date of birth |  | Weight (kg) |  |

| Do you or have you ever worn an electronic device?  (e.g. Pacemaker, Neuro stimulator, Pain-, Insulin pump, Cochlear Implant) | Yes | No |
| --- | --- | --- |
| Do you have any implants or prosthesis (e.g. Cochlear Implant, Artificial heart valve, Hip prosthesis, Insulin pump, etc.)? | Yes | No |
| Do you have metal pieces or fragments in your eye (Shrapnel wounds, Operation clips, Piercing, etc.)? Do you work in metal works? | Yes | No |
| Have you ever had a metal splinter in your eye?  If yes:  Did you go to a doctor?  Did the doctor insure that all the metal was removed? | Yes  Y Y | No  No  No |
| Do you have a hearing aid or removable teeth prosthesis? (Please remove them for simulation and treatment) | Yes | No |
| Have you ever had an operation in your head, eye or heart? What kind of operation? Was something implanted? | Yes | No |
| Do you have any tattoos or permanent makeup? | Yes | No |
| Can you lie still for an extended period of time?  (e.g. Due to pain, trembling, sneezing or coughing?) | Yes | No |
| Do you have a transdermal patch? (ex: Pain, Nicotine, Diabetes) | Yes | No |
| Are you claustrophobic? | Yes | No |

**I confirm, that I have truthfully answered the questions above. I agree with the proposed MR-Linac treatment**

Place, Date: Signature:

**Questionnaire verified , Patient is MR approved** Signature:

Supervising Physician

Please ensure that before entering the MR bunker that you have removed watches, jewelry, wallets, and any other metallic object. Credit cards can be delated and metal objects can cause harm to yourself and to others.

# Risk analysis

List of evaluated risks in implementation of online adaptive MRgRT

I= Incidence, S=Significance, D=Detection, N=Number

| **Risk**  **category** | **Potential mistake** | **Consequence** | **Reason** | **Control measures** | **I** | **S** | **D** | **N** |
| --- | --- | --- | --- | --- | --- | --- | --- | --- |
| patient  setup | Suboptimal positioning of patient or coils | Poor MR Quality, Body not recognized, wrong dose calculation | Coils setup incorrect longitudinally | Coils are positioned after setup in Laser-system. Coil center is centers on laser too | **1** | **7** | **3** | **21** |
| patient  setup | Suboptimal positioning of patient or coils | Poor MR quality, distortion in target volume, geometrical miss of the target up to 3mm possible | Distance isocenter to PTV >8cm or Distance Isocenter to coil too big | At simulation, pictures of setup are taken and table position is saved. RTTs have this information on a tablet-PC in room | **3** | **3** | **2** | **18** |
| Imaging | Parts of Body not in FoV | Body structure incorrect, thus dose calculation incorrect | FoV wrong at setup scan | FoV is daily adapted on the anatomy. Control on registry to the original-MRI by RTT performing registration. | **4** | **3** | **1** | **12** |
| Imaging/  registration | Fusion incorrect | GTV at wrong place, GTV has to be recontoured, risk of size changes, longer table time | Fusion incorrect and/or not on GTV | RTT registers, physician checks fusion – 4-eyes-primciple | **4** | **2** | **1** | **8** |
| Imaging/  registration | CT incorrect deformed to MRI | CT density incorrect, dose calculation incorrect | Autofusion does not work optimal | CT-density-map is checked by RTT and physics after contouring (4-eyes). Control of effective depth in 2cm (Original plan) | **4** | **2** | **2** | **16** |
| Imaging/  registration | Final copy contour forgotten | Rigid structures are on the wrong position, all contours have to be recontoured, longer table time | One step in workflow has been forgotten | When it is noticed on contouring, go back to registration (time loss 2 minutes) | **3** | **1** | **1** | **3** |
| contouring | Contour rules wrong or missing | Optimizing does not work as requested | Rules were either not generated or in the wrong order | Rules are checked on plan approval and additionally physics performing adaptation checks the physical-PTV | **3** | **1** | **3** | **9** |
| contouring | CTV Definition incorrect | Physician does not know the original CTV and defines anew on the adaptive MRI. Thus, change in PTV to an incorrect PTV. | CTV description not clear in patient’s record. | Contouring physician describes CTV-definition in patients Journal.  Adapting physician checks initial CTV in Eclipse. CTV-sizes is compared daily with the baseline CTV. | **2** | **3** | **3** | **18** |
| contouring | OAR incorrect | Plan suboptimal | Deformable registration incorrect, physician did not correct manually. | Checklist item added: “OAR in Ring (2cm around PTV) adapted” | **2** | **2** | **2** | **8** |
| contouring | Incorrect override of water/air | dose calculation incorrect | Structures were not adapted correctly during adaptive contouring/planning | RTT and Physics check density map after contouring | **4** | **2** | **2** | **16** |
| contouring | Forgotten Post-processing of contours | Without processing poor plan optimization. Possibly plan unacceptable, patient can’t be treated | Post-processing of contours were forgotten | Checklist item «post-processing done» | **3** | **1** | **2** | **6** |
| contouring | Skin contour incorrect | dose calculation incorrect | MR bad quality or registration error | Checklist item «Skin contour correct», additionally comparison of equivalent depth in 2cm of original plan | **3** | **7** | **2** | **42** |
| contouring | Visual control of densities forgotten | dose calculation incorrect | MR bad quality or registration error | Checklist item «density correct», additionally comparison of equivalent depth in 2cm of original plan | **3** | **7** | **2** | **42** |
| contouring | GTV increases slowly | GTV growing from adaptation to adaptation, finally treatment of bigger volume | GTV is drawn bigger «on the fly» than upfront without time pressure. | Always Start from original baseline plan. Daily comparison of GTV size tot he baseline plan | **4** | **2** | **2** | **16** |
| adaptive planning | Incorrect electron densities | dose calculation incorrect | Assessed wrong manually | Checklist item «density correct», additionally comparison of equivalent depth in 2cm of original plan | **2** | **4** | **2** | **16** |
| adaptive planning | Doubled normalization | Verification system calculates wrong dose. Aberration is too big for primary system; patient can’t be treated with the plan. | Normalization is pressured twice, for test of different normalizations | Checklist and detailed procedures | **2** | **1** | **1** | **2** |
| adaptive planning | Optimal dose distribution not reached | Patient is treated with suboptimal plan or has a treatment interruption | Patients anatomy significant changed to simulation | All significant decline in plan quality are evaluated offline for finding the source/reason. | **2** | **2** | **1** | **4** |
| adaptive planning | Slightly worse plans | New plan is used daily as new baseline plan. Thus, small changes from plan to plan could mask big changes from the initial baseline plan. | Slightly worse plan is accepted every day due to time pressure. | Dose constraints are set fix for all measures. Additional parameter for daily control of conformity. In general baseline plan is used for all adaptions | **5** | **2** | **2** | **20** |
| adaptive planning | New plan lost | «Treat with old Plan» is set by mistake. New plan is deleted and whole workflow has to be repeated. | Adherence to checklist step by step. Set «treat with new plan”. | Checklist and detailed procedures | **2** | **2** | **1** | **4** |
| QA | Online QA missing | Patient is closed by mistake before online-QA was performed. This is not possible retrospectively. Patient can’t be irradiated. | Missing checklist and setting «treat with new plan» before online QA was done | Checklist and detailed procedures | **2** | **2** | **1** | **4** |
| QA | QA misses standard goals | QA not successful, adaptive plan can’t be applied | Mistake on QA-process, Mistake in Planning | Checklist and detailed procedures | **2** | **2** | **1** | **4** |
| QA | Wrong patient chosen for QA | Patient is treated without QA | Inattention | Patients name is displayed in control software. RTT confirms to physics patients correct name | **1** | **2** | **5** | **10** |

Planning characteristics per treatment technique

|  | SBRT (n=94) | Mixed treatment (boost at the MRIdian) (n=17) | Conventional fractionation (n=13) |
| --- | --- | --- | --- |
| Respiratory motion compensation strategy   - Breath-hold n (%)   - Insp n (%)   - Exp n (%) | 58 (62)  13 (22)  45 (78) | 4 (24)  0 (0)  4 (100) | 2 (15)  1 (50)  1 (50) |
| Number of fractions  median (range) | 4 (1-15) | 3.5 (3-6) | 35 (20-35) |
| Treatment duration in days; median (range) | 7 (1-15) | 5 (3-12) | 49 (29-52) |
| Dose per fraction in Gy  median (range) | 7 (4.25 – 25) | 2.2 (1.8-3) | 2 (2-3) |
| Inhomogeneous irradiation  n (%)   - @ 65% - @ 80% - @ 85% | 72 (77)  48 (67)  22 (30)  2 (3) | 0 | 0 |
| Online adaptive planning  n (%) | 77 (82) | 14 (82) | 0 |

# Special aspects of treatment sites

## Liver

Liver SBRT was performed in 17 patients with in total 19 treatment courses. One patient was treated with 3 courses for in total 7 liver metastases. All but one patient treated with SBRT for liver had liver metastases. This one patient suffered from perihilar cholangiocarcinoma and was re-irradiated at the MR-LINAC for local progression 17 months after the first irradiation. All courses were treated mono-isocentric. In 3 courses, more than 1 volume was treated: in 2 courses 2 metastases were treated with the same plan and in one case even four volumes were treated with one treatment plan and isocenter.

As outlined above, in total 4 patients were treated for their boost of a cholangiocellular carcinoma at the MR-LINAC. Beside of the general advantages of MRgRT already described in the introduction section, the MR-sequence of the MR-LINAC system seems to be at least comparable to diagnostic MR-imaging modalities in terms of visibility and definition of the tumor in this specific entity. See Figure 1.


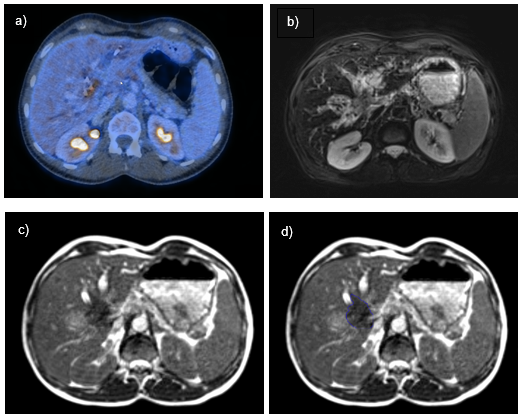


Fig 1 Appearance of a cholangiocarcinoma in FDG-PET/CT (a), diagnostic MRI (b) and simulation image in the MR-LINAC (c) with the tumor delineated in blue (d)

## Bone

Ten Patients with symptomatic non-spine metastases were treated with one fraction of high dose SBRT in a one-day workflow with immediate on-table contouring and treatment planning without previous planning-CT. This allowed a planning and treatment within an hour, without repeated potentially painful positioning.

## Prostate/Pelvis

We performed SBRT for prostate cancer in 9 patients. These patients were able to obtain a SBRT in 5 fractions with single doses of 7.25 Gy without need for implantation of transponders into the prostate.

Eight patients with locoregional macroscopic recurrence had a mixed treatment in their salvage situation. They received a boost of total 6 Gy in 2 Gy single doses to the GTV with daily o-ART, additionally to the usual radiation to the prostate bed with 66 Gy in 2 Gy single doses. This allowed a target dose of 72Gy with maximum sparing of critical structures like rectum, anastomosis and bladder neck. One patient with prostate cancer was treated hypofractionated at the MR-LINAC in 20 fractions due to an unfavorable anatomy with a lot of small bowel surrounding the GTV.

Thirteen cases with nodal recurrences in the pelvis were treated with SBRT allowing maximum bowel sparing.

## Head and neck

Until now, HNC not a usual indication for MRgRT [1]. The patients with a full course included here are all treated in a prospective trial (MARTHA) with narrow margins, online gating and weekly offline adaptation with the aim of maximal sparing of the parotids (ClinicalTrials.gov Identifier: NCT03972072). Our initial concerns of possibly enhanced claustrophobia with flexible coils over thermoplastic masks and bore were not confirmed. The mask was tolerated equally as in the conventional LINAC – accordingly, there were no drop-outs due to mask concerns or claustrophobia.

# Quality assurance of the machine

A comprehensive machine QA program was implemented based on national QA guidelines for linear accelerators, international guidelines for A of MR scanners and some additional checks to check the interplay between the MR scanner and the linear accelerator [2, 3].

In general, all beam parameters were stable over the first year of operation. In Fig. 2 the dose output of the 6FFF linear accelerator measured with a PTW Farmer Chamber (PTW Freiburg, Type TW30013) in a solid water phantom. In the beginning, the output was measured daily with the Farmer chamber until August 2019 and afterwards monthly with the Farmer chamber. Over the first year a maximum deviation of 1% (the baseline was set after reference dosimetry in a water tank) was observed.


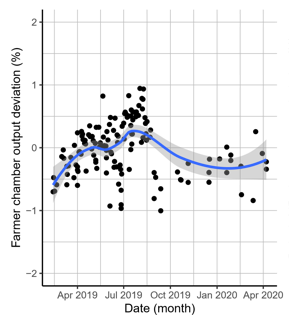


Fig. 2 dose output of the 6FFF linear accelerator measured with a PTW Farmer Chamber (PTW Freiburg, Type TW30013) in a solid water phantom

The frequency of conducting regularly imaging QA procedure is higher in our clinic as for MRI scanner in radiology departments since the positioning of the patient and gating of relevant structures during the treatment depends highly on the performance of the imaging quality and a reliable stable MRI isocenter. Three different QA imaging phantoms are used for our weekly extended imaging QA program as recommended in the AAPM Report No 100 [2]: the ACR (American College of Radiology), a spherical NEMA (National Electrical Manufacturers Asssociation) and a SI (Spatial Integrity) phantom.

The ViewRay MRIdian system has no direct access to the geometrical isocenter and no additional imaging modality to verify the position of QA phantoms or water tanks. A laser isocenter which is axial shifted by 155cm is used to position QA phantoms outside of the bore of the treatment machine. A frequent check to verify the correlation between the imaging, laser and radiation therapy isocenter is essential to ensure a safe treatment. A starshot film measurement with a dedicated QA phantom (five beams) is conducted weekly, where the correlation of the three isocenters is tested. We achieved a diameter of less than 1mm for the smallest intersecting circle of the RT isocenter and less than 1mm deviation to the MRI and laser isocenter. Fig. 3 shows the transversal offset of the MRI vs RT isocenter.

Fig. 3 show the transversal offset of the MRI vs RT isocenter

**References**

[1.] Boeke S, Mönnich D, van Timmeren JE, Balermpas P: MR-Guided Radiotherapy for Head and Neck Cancer: Current Developments, Perspectives, and Challenges. Frontiers in oncology 2021, 11:616156.

[2.] Jackson EB, MJ; Drost, DJ, Och, J, Pooley, RA, Sobol, WT, Clarke, GD: AAPM Report No. 100. Acceptance Testing and Quality Assurance Procedures for Magnetic Resonance Imaging Facilities. In.; 2010: 1-38.

[3.] Quality Control of Medical Electron Accelerators. SSRMP Recommendation Nr. 11. In.: Swiss Society of Radiobiology and Medical Physics 2014.
